# Annual distributions of insecticide-treated nets to schoolchildren and other key populations to maintain higher ITN access than with mass campaigns: a modelling study for mainland Tanzania

**DOI:** 10.1186/s12936-022-04272-w

**Published:** 2022-08-26

**Authors:** Hannah Koenker, Matt Worges, Benjamin Kamala, Peter Gitanya, Frank Chacky, Samwel Lazaro, Charles Dismas Mwalimu, Sijenunu Aaron, Deodatus Mwingizi, David Dadi, Ato Selby, Naomi Serbantez, Lulu Msangi, Dana Loll, Joshua Yukich

**Affiliations:** 1 USAID Tanzania Vector Control Activity, Tropical Health, Baltimore, MD USA; 2 USAID Tanzania Vector Control Activity, Tropical Health, New Orleans, LA USA; 3 USAID Tanzania Vector Control Activity, Johns Hopkins University School of Public Health Center for Communication Programmes, Dar es Salaam, Tanzania; 4 National Malaria Control Programme, Ministry of Health, Dodoma, Tanzania; 5 US President’s Malaria Initiative, Dar es Salaam, Tanzania; 6 USAID Tanzania Vector Control Activity, Johns Hopkins University School of Public Health Center for Communication Programs, Baltimore, MD USA

**Keywords:** ITN, Malaria control, Vector control, Insecticide-treated net, Bed net, Tanzania, Quantification, School children, Distribution

## Abstract

**Background:**

Since 2013, the National Malaria Control Programme in mainland Tanzania has deployed annual distributions of insecticide-treated nets (ITNs) through primary schools to maintain ITN access and use. This School Net Programme (SNP) is slated to be used throughout mainland Tanzania by 2023. This modelling study projects ITN access under different ITN distribution strategies and quantification approaches.

**Methods:**

A stock and flow model with a Tanzania-specific ITN decay rate was used to calculate annual net crops for four different ITN distribution strategies, varying quantification approaches within each strategy. Annual nets-per-capita (NPC) was derived from net crop and a standardized population projection. Nonparametric conditional quartile functions for the proportion of the population with access to an ITN (ITN access) as a function of NPC were used to predict ITN access and its variability. The number of ITNs required under the varying quantification approaches for the period 2022–2030 was calculated.

**Results:**

Annual SNP quantified using a “population times 15%” approach maintained ITN access between 80 and 90%, when combined with reproductive and child health (RCH) ITN distribution, requiring 133.2 million ITNs. The same strategy quantified with “population times 22%” maintained ITN access at or above 90%, requiring 175.5 million ITNs. Under 5-year mass campaigns with RCH distribution for pregnant women and infants, ITN access reached 90% post-campaign and fell to 27–35% in the 4th year post-campaign, requiring 120.5 million ITNs over 8 years. 3-yearly mass campaigns with RCH reached 100% ITN access post-campaign and fell to 70% in the 3rd year post-campaign, requiring 154.4 million ITNs.

**Conclusion:**

Given an ITN retention time in Tanzania of 2.15 years, the model predicts that mass campaigns conducted every 3 years in mainland Tanzania will not maintain ITN access at target levels of 80%, even with strong RCH channels. Mainland Tanzania can however expect to maintain ITN access at 80% or above by quantifying SNP using “population × 15%”, in addition to RCH ITN delivery. This strategy requires 14% fewer ITNs than a 3-year campaign strategy while providing more consistent ITN coverage. Meeting the targets of 80% ITN use would require maintaining 90% ITN access, achievable using a “population times 22%” quantification approach for SNP.

**Supplementary Information:**

The online version contains supplementary material available at 10.1186/s12936-022-04272-w.

## Background

The burden of malaria in Tanzania is substantial, with six million cases in 2020 [1]. The National Malaria Control Programme (NMCP) has distributed insecticide-treated nets (ITNs) since 2004, and along with improved case management, prevention of malaria in pregnancy, and IRS, have contributed to declines in malaria incidence from 162 per 1000 population in 2015 to 106 in 2020 [2] on mainland. Mainland Tanzania is unique in implementing large-scale school-based ITN distribution as a primary channel in over half the country [3]. Annual distributions of ITNs through primary schools, known as the School Net Programme (SNP) began in 2013. SNP was scaled up from three regions in 2013, 2014, and 2015, to seven regions in 2016, and to 14 of 26 mainland regions from 2017 onward [4]. The Tanzania National Voucher Scheme (2004–2014) delivered coupons redeemable for ITNs (with a small copay) to pregnant women at their first antenatal care clinics (ANC) attendance and to infants at first measles immunization visits, known as IVD (immunization and vaccine development). In 2016, free distribution to these groups commenced. The NMCP conducted four mass campaigns: for children under five in 2007–8 [5], a universal coverage campaign in 2010–11 [6], a mass replacement campaign in 2015 (in 23 of 26 mainland regions), and most recently in 2020, targeting 50 councils in 12 regions.

The mainland Tanzania ITN strategy aims to use SNP as the primary distribution channel, with reproductive and child health (RCH) providing nets to biologically vulnerable groups. Mass campaigns are deployed on a targeted basis, triggered only when the proportion of the population with access to an ITN (ITN access) falls below 40%, to quickly scale up coverage to target levels. Additional channels for other vulnerable groups including the elderly and people living with HIV are planned.

Quantification for SNP has previously relied on NetCALC modelling [3] at national as well as regional level, or on simplified best estimates of what number of ITNs might be needed to achieve coverage goals. Recent work has highlighted the difficulties programmes face in reaching universal coverage targets [7, 8] but have focused on mass campaign strategies, which face inherent inefficiencies due to oversaturation of ITNs within some households while others remain under-supplied [9, 10].

Quantification approaches for future years of continuous distribution in mainland Tanzania would benefit from a simplified population-based algorithm that is informed by the distribution performance of the SNP over the past nine rounds, the distribution performance of the RCH channel introduced in 2016 (Koenker et al. in preparation), and by recent research into country-specific ITN retention times [8]. A data-driven but easily conceptualized and implementable approach is needed for annual school-based distribution strategies that are implemented alongside routine ITN delivery through health clinics.

This paper describes the results of the modelling across four different ITN distribution strategies, the implications of different quantification approaches, and provides recommendations to meet targets for ITN access and use.

## Methods

**Fig. 1:**
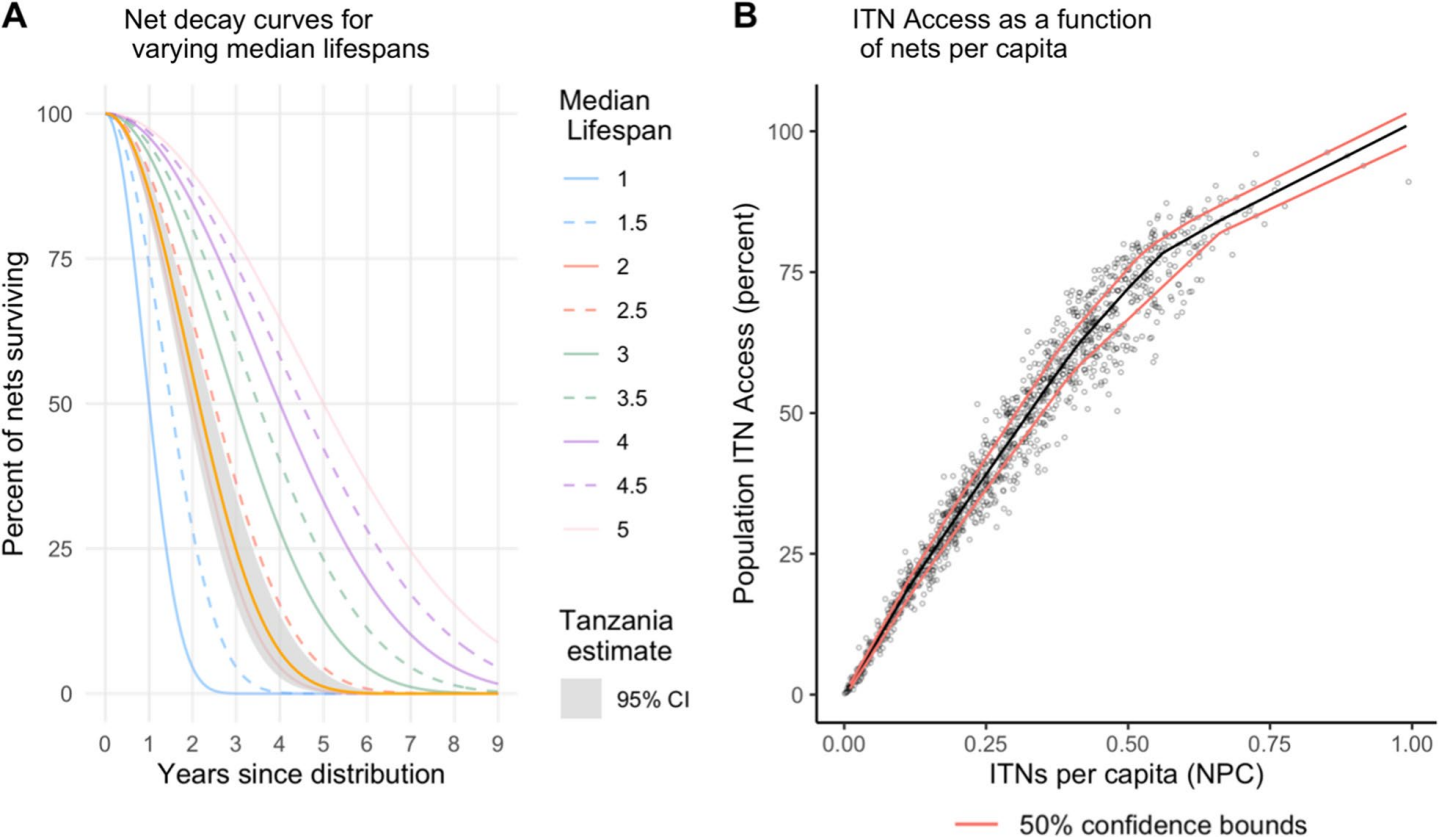
**A** Net survival decay curves for varying median lifespans, with Tanzania's estimated median lifespan shown in orange with its 95% CI. **B** Population ITN access vs ITNs per capita from 124 Demographic and Health Surveys and Malaria Indicator Surveys. Black line indicates the nonparametric conditional quartile fit, with red lines indicating the 50% confidence bounds

An age-structured stock and flow model was used to generate estimates of ITN access [8]. Within the stock and flow model, distributed ITNs were decayed annually using an estimated Tanzania specific median lifespan of 2.15 (CI 1.88–2.43) from Bertozzi-Villa et al. [8]. The net decay rates rely on a smooth-compact loss function developed by Nakul Chitnis and described in Koenker et al. and Bhatt et al. [3, 9], shown in Fig. 1 Panel A [11][3]. The decay rate equation is provided in the Additional file 1.

Commonly deployed ITN distribution strategies were described in four scenarios, varying the population-based quantification factor (and thus the quantities of ITNs ‘delivered’) within each scenario. The number of ITNs was set equal to the population multiplied by the quantification factor, ranging from 8–25% for school distribution. ITNs for mass campaigns were quantified using population divided by 1.8 recommended by the World Health Organization (WHO).

For routine distribution, ITNs were quantified using “population multiplied by 7%” for scenarios 3 and 4 and varied between 5 and 7% for scenarios 1 and 2. Pregnant women are generally around 4–5% of the population and children under 1 year comprise 3–4% of the population. The number of ITNs distributed to pregnant women and infants in Tanzania is equivalent to 7% of the population over the past 3 years (Koenker et al. in review).

The following ITN distribution scenarios were used to calculate annual net crops, defined as the total ITNs surviving each year from prior distributions and new distributions:

1. Mass campaigns every 5 years with continuous distribution of RCH (ANC and IVD) ITNs, quantifying ITNs for RCH channel at population multiplied by 5%, 6%, and 7%.
2. Mass campaign every 3 years with continuous distribution of RCH (ANC and IVD) ITNs, quantifying ITNs for RCH channel at population multiplied by 5%, 6%, and 7%.
3. Mass campaign every 3 years with continuous distribution of RCH (ANC and IVD) ITNs fixed at 7% and quantifying ITNs for annual SNP in the years between campaigns using population multiplied by 5%, up to population multiplied by 13%, in increments of 1%
4. Full-scale annual SNP with ITNs quantified using population multiplied by 0%, up to population multiplied by 25%, in increments of 1%, along with continuous distribution of RCH (ANC and IVD) ITNs fixed at population multiplied by 7%.

All scenarios began in 2022 and ended in 2030. ITN access and net crop for the start year was calculated separately using the same model, inputting the historical ITN distributions in mainland Tanzania and applying the decay rate to each annual crop of nets; this work is described elsewhere (Koenker et al. in preparation). While Tanzania has implemented SNP in 14 regions and mass campaign in the other 12 mainland regions in 2020, an overall average net crop and ITN access were calculated as the starting point for all scenarios. Scenarios 1–3 assumed a mass campaign in 2023 quantified using the WHO recommended population/1.8 [12]. Scenario 4 continued annual distributions from 2022. The projected population of mainland Tanzania from the National Bureau of Statistics was used as the denominator for population-based indicators.

All surviving nets from the various channels were summed for each year and council to create a total net crop. Net crop was divided by the projected population to provide nets-per-capita (NPC) in each year and council. A nonparametric conditional quartile function for ITN access as a function of NPC was estimated using data from 124 Demographic and Health (DHS) and Malaria Indicator Surveys (MIS), and subsequently used to predict ITN access as a function of the stock and flow model prediction of NPC at the council level (Fig. 1 Panel B). Uncertainty was propagated using sensitivity analysis to establish best- and worst-case estimates of ITN access given the confidence intervals for both estimated median lifespan and the function of ITN access vs NPC.

## Results

**Fig. 2:**
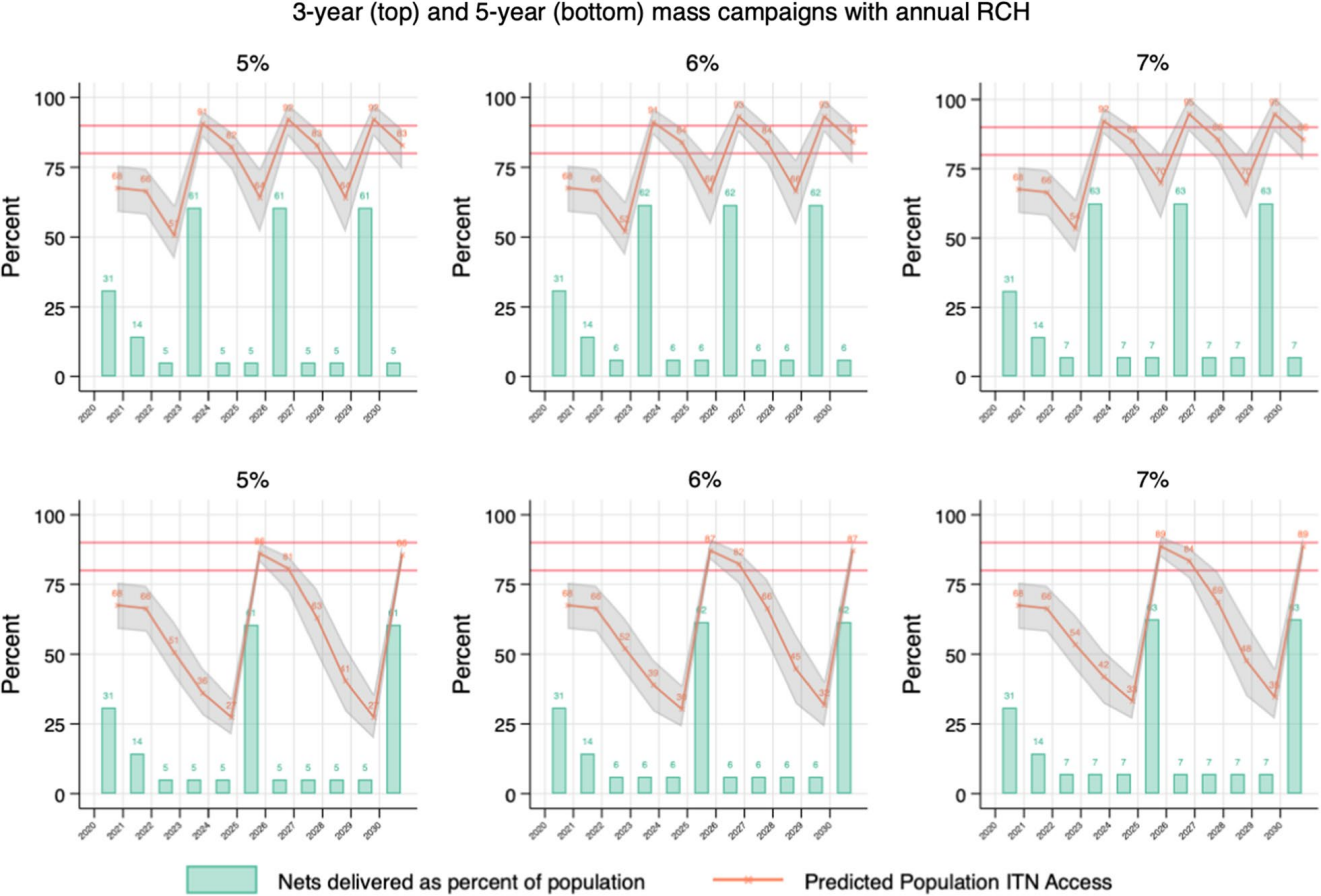
Projected ITN access for Tanzania mainland, with mass campaigns implemented every 3 years (top row) or every 5 years (bottom row) with varying performance levels of RCH distribution (ITNs equal to 5–7% of the population)

**Fig. 3:**
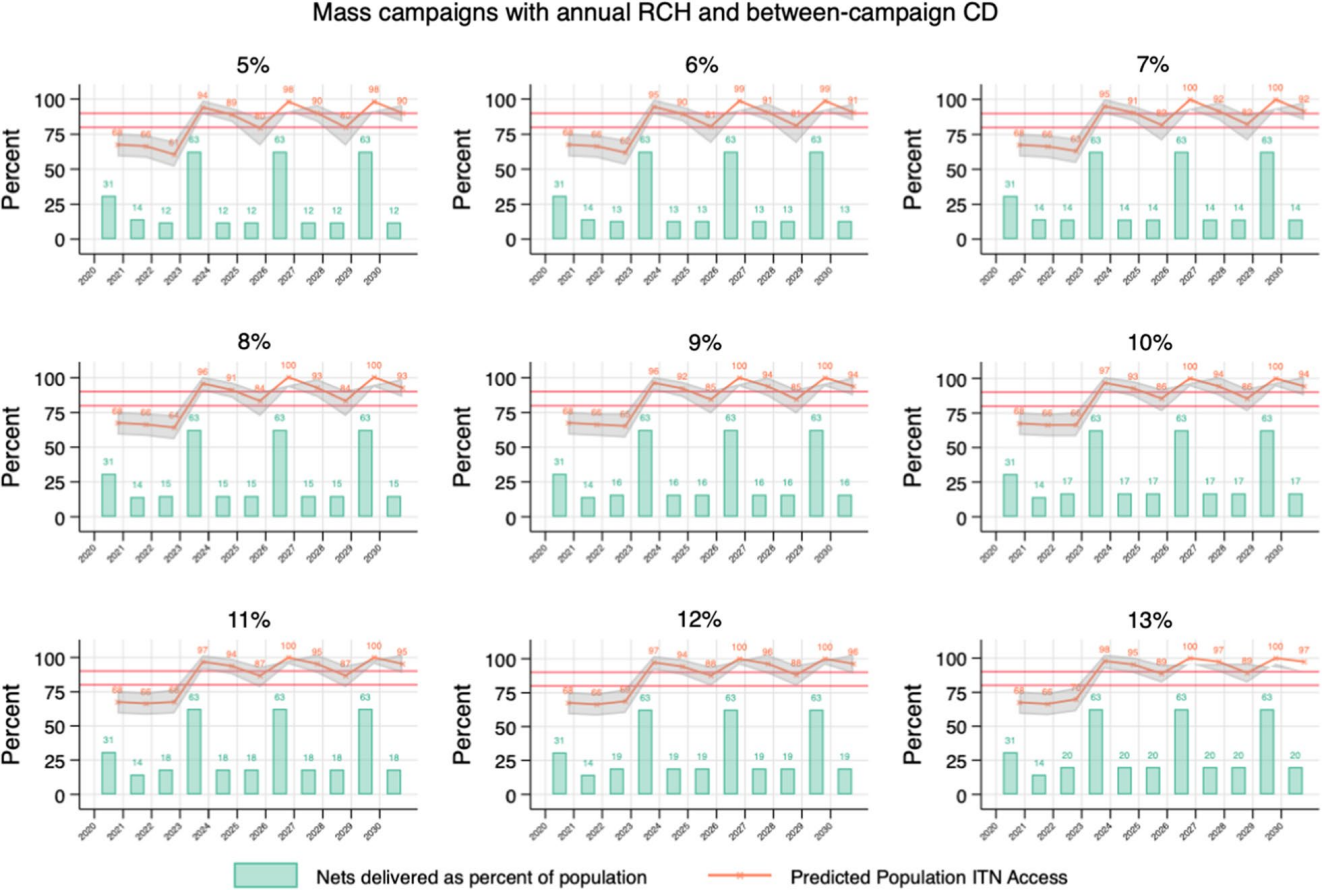
Projected ITN access for Tanzania mainland if 3-year mass campaigns are implemented in 2023, 2026, and 2029, and with RCH distribution assumed at ITNs equal to 7% of the population, and varying intensity of annual school distributions (ITNs equal to 5–13% of the population)

**Fig. 4:**
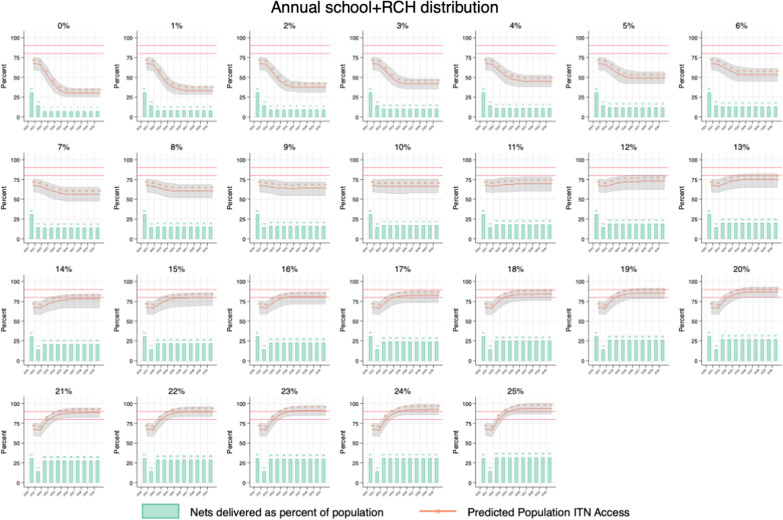
Projected ITN access under varying levels of annual school distribution (ITNs equal to 0–25% of population). RCH is fixed at 7%

Full results of the scenario modeling are shown in Fig. 2, Fig. 3, and Fig. 4. For the simple 3-year mass campaigns scenario, predicted ITN access rose to 90% immediately following each campaign, then fell to between 63%, 66%, and 70% at levels of RCH ITN distribution equal to 5%, 6%, and 7% of the council population, respectively. When campaigns were done every 5 years (mainland’s de facto schedule since 2010), ITN access fell to 27–35% prior to the subsequent campaigns.

In scenario 3 (mass campaigns with RCH and between-campaign school or community distribution), RCH inputs were held steady at 7% of the council population, reflecting the 2021 delivery rate of ITNs to pregnant women and infants across mainland Tanzania. Predicted ITN access reached 100% immediately following each campaign and declined to 80% in the 5% annual distribution case, and to 90% in the 13% case.

In the fully continuous scenario (#4), RCH was again fixed at 7% of the council population, and annual school distributions were input, quantifying ITNs for each year by multiplying the population by 0% in the first run, increasing the quantifier in increments of 1% for subsequent runs, up to 25% annually. With no school distribution and only RCH, predicted ITN access fell quickly and stabilized at 30% by 2025. At population times 10%, predicted ITN access stabilized at 68%. Only at population times 15% did predicted ITN access stabilize at target levels of 80%. At population times 22%, predicted ITN access stabilized at 90%.

**Fig. 5:**
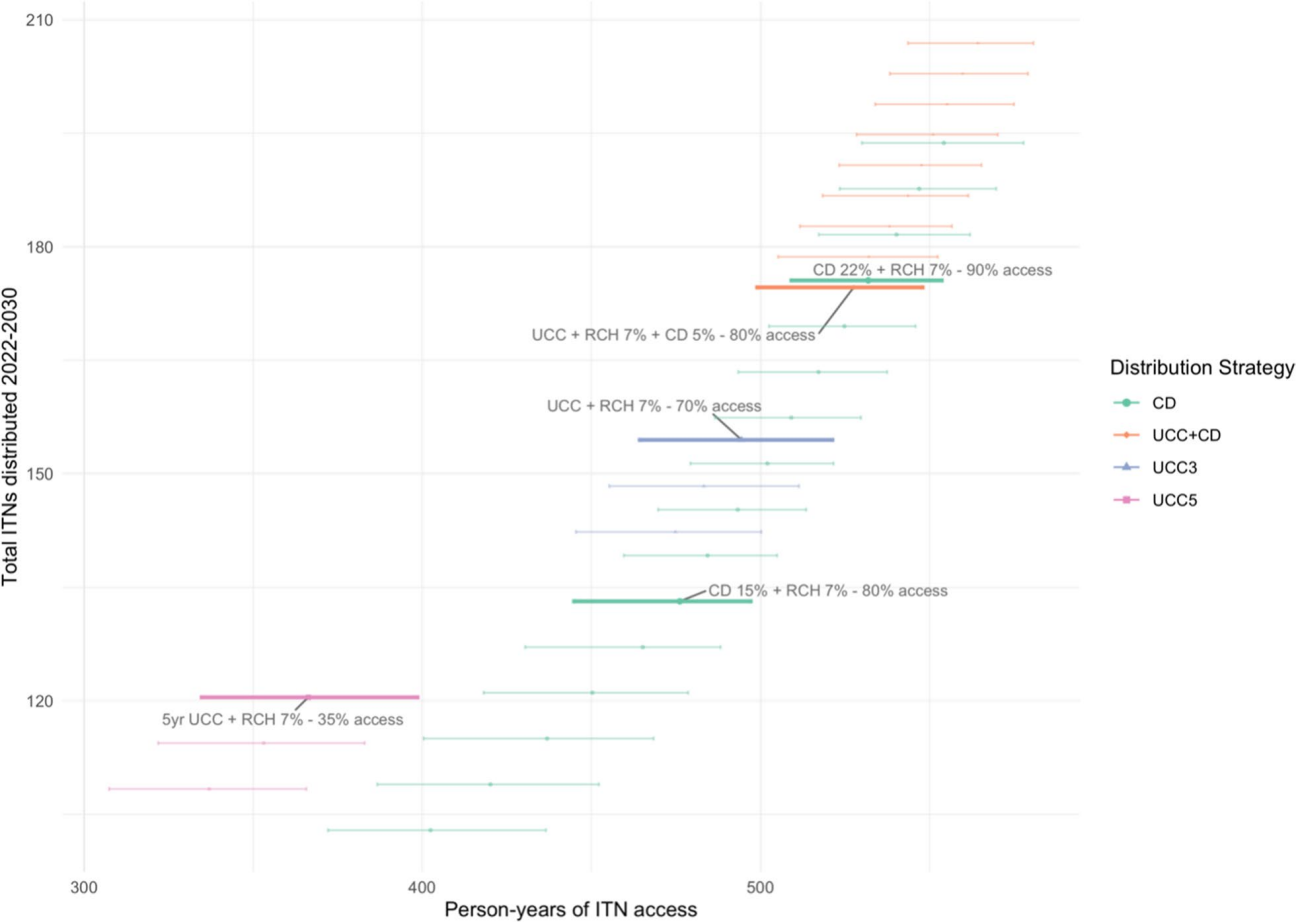
Total ITN need 2022–2030 and total person-years of ITN access

The numbers of ITNs required under different ITN distribution scenarios, aiming to maintain ITN access at or above 80% where possible within each scenario, are summarized in Table 1 and Fig. 5.

**Table 1:** ITNs required under different distribution scenarios with estimated ITN access achieved over 2022–2030

ITN distribution scenario	Quantification	Max ITN access	Min ITN access	Total ITNs required 2022–2030 mainland	% difference in net need vs 3 year campaigns	Person-years of ITN access
5-year mass campaigns + RCH	Campaign = population/1.8 RCH = population × 7%	90	35	120.5 m	− 22%	753
3-year mass campaigns + RCH	Campaign = population/1.8 RCH = population × 7%	100	71	154.4 m	Ref.	881
3-year mass campaigns + RCH + school between campaign years	Campaign = population/1.8 RCH = population × 7% School = population × 5%	100	80	174.6 m	+ 13%	914
4a. RCH + school targeting minimum 90% ITN access	RCH = population × 7% School = population × 22%	94	90	175.5 m	14%	918
4b. RCH + school targeting minimum 80% access	RCH = population × 7% School = population × 15%	90	80	133.2 m	− 14%	863

Over the period 2022–2030, mainland Tanzania would require roughly 154.4 million ITNs with a mass campaign plus 7% RCH strategy, and 174.6 million ITNs for mass campaign plus 7% RCH plus 5% school distribution. The former strategy would reach minimum levels of 71% ITN access between campaigns, and the latter minimum levels of 80%. The full-scale school distribution with RCH at 7% and school distribution at population × 22% would maintain ITN access between 90–94%, requiring the same number of nets as the campaigns + schools option, but with more stable coverage. Finally, ITN access could be maintained between 80 and 90% with RCH at 7% and school distribution at 15%, requiring only 133.2 million ITNs over 8 years, a savings of 14–25% over other options. The 5-year campaign strategy, while requiring the least amount of ITNs, also results in severe declines in ITN access between campaigns, demonstrating the impact that campaign delays can have on ITN coverage.

## Discussion

The model for projecting ITN access employed in this study showed that 80% ITN access could be maintained with a continuous ITN programme that combines routine ITN distribution with school-based distribution, with fewer nets required and higher person-years of ITN access, compared to the typical ITN strategy of mass campaigns every 3 years and routine distribution. With RCH ITNs currently reaching 7% of the population, school ITNs would be separately quantified each year by multiplying the population by 15%. This same quantification can also be split across several different distribution channels, as NMCP is currently planning, to better reach additional vulnerable groups including people living with HIV/AIDS and the elderly. This option requires 14% fewer ITNs over the same 8-year period compared to a series of mass ITN distribution campaigns conducted once every 3 years combined with RCH distribution. At the same time, it provides higher and more stable coverage over time (between 80 and 90% population ITN access).

Since the beginning of the SNP, ITN quantification has been a complex and cumbersome process [3]. Using an evidence-based population-multiplied-by-X quantification approach is desirable to simplify the process of forecasting procurement needs. These findings are supported by retrospective research in Tanzania that found that continuous distribution at quantities equal to population × 20–25% led to higher ITN access in household surveys than when quantified at lower levels (Koenker et al. in review). School distribution in Tanzania has previously been demonstrated to maintain population ITN access and use in the absence of mass campaigns [4, 13].

The estimates of ITN access in this analysis are highly sensitive to the decay rate for nets. If ITNs were retained for longer periods, fewer ITNs would be needed than presented here. While it has been demonstrated that social behaviour change interventions can improve net care behaviours, leading to longer median lifespan [14], it is unclear whether such interventions can be successful at scale. More durable ITNs may also last longer on average, similarly affecting total ITN need [15], although durability has also been shown to vary greatly by location and primarily due to behaviors rather than product characteristics [16]. While these recommended quantification approaches are relevant for Tanzania, similar scenario testing can be used to identify appropriate quantification approaches for other countries, using country-specific decay rates.

ITN distribution strategies targeting primary school students may have additional benefits in reducing the ITN access gap for school-age children, which is the leading cause of non-use of ITNs in this group [4, 17–21]. School-age children have high rates of asymptomatic parasitaemia and may contribute significantly to malaria transmission [22, 23]. Improving older children’s access to ITNs and thus their ability to sleep under a net consistently could lessen the burden of disease in this group, improving school attendance and performance, and contribute to reduced transmission overall.

Finally, it must be noted that the targets for ITN coverage rely on ITN use as the primary indicator. If the target is for 80% ITN use to be achieved, ITN access targets would need to be maintained at 90–95%, given no one can use an ITN if they do not have access to one within their household and ITN use is typically 85–90% of ITN access in Tanzania [8, 24, 25]. The quantification approach to maintain 90% ITN access would be population × 29% annually (7% from RCH and 22% from SNP) and would require 25% more ITNs than maintaining access at 80%. It will be crucial to model whether the cost of achieving these targets provides sufficient incremental health benefit, to inform global and national-level policy decisions.

## Conclusion

Given an average net lifespan of 2.15 years, NMCP Tanzania should consider quantifying annual school distributions using “population times 15%” in addition to current high levels of RCH ITN delivery to meet the targets of 80% ITN access, or population times 22% for targets of 90% access and 80% ITN use.

## Supplementary Information

**Additional file 1**: Net decay formula.

## Data Availability

ITN delivery data are available upon request from the NMCP. Population estimates are available upon request from the Tanzania National Bureau of Statistics.
