# Supplementary material for: Annual distributions of insecticide-treated nets to schoolchildren and other key populations to maintain higher ITN access than with mass campaigns: a modelling study for mainland Tanzania
Source: Malar J. 2022 Aug 26;21:246. doi: 10.1186/s12936-022-04272-w (PMC9417077; doi:10.1186/s12936-022-04272-w)
Supplement: Supplementary file 1 — Additional file 1: Net decay formula. [file 12936_2022_4272_MOESM1_ESM.docx]

# **Additional file 1:**

Net decay formula:

Where *c_n_* = net crop in the starting year, *c_n+t_* = net crop in a subsequent year, k = the constant 20, *t* is the number of years since the net distribution, and *L* is the value 11.75, representing a median lifespan of 2.15 years.

$$c_{n+t}= c_{n}\times\exp\left( k-\frac{k}{1- \left( \frac{t}{L} \right)^{2}} \right)$$
